# Supplementary material for: Molecular Cloning and Characterization of a Serotonin N-Acetyltransferase Gene, xoSNAT3, from Xanthomonas oryzae pv. oryzae
Source: Int J Environ Res Public Health. 2023 Jan 19;20(3):1865. doi: 10.3390/ijerph20031865 (PMC9914633; doi:10.3390/ijerph20031865)
Supplement: Supplementary file 1 [file ijerph-20-01865-s001.zip › ijerph-2145815-supplementary.pdf]

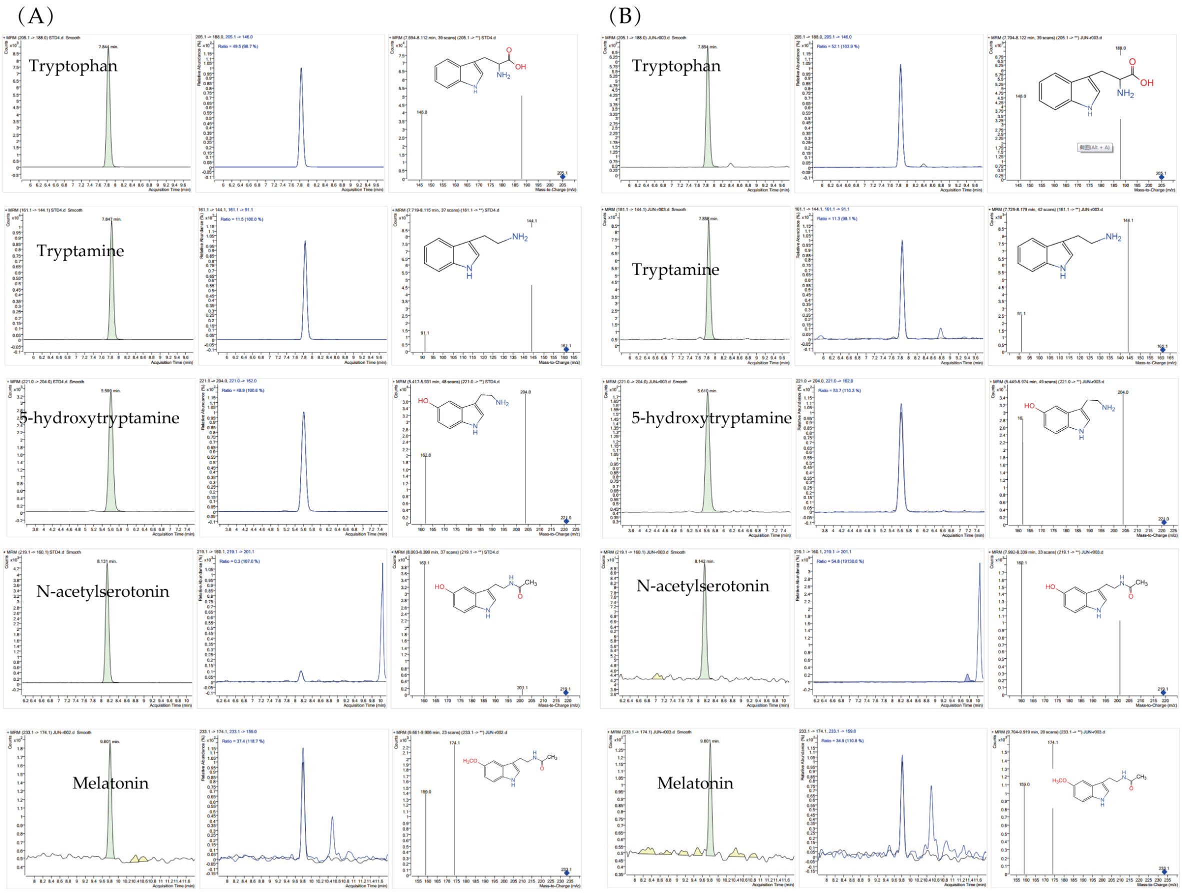


**Figure S1:** Detection of MT biosynthetic intermediates in Xoo. (A) Chemical structures and chromatograms of tryptophan, tryptamine, 5-hydroxytryptophan, 5-hydroxytryptamine, N-acetylserotonin and MT standards. (B) Chromatograms of tryptophan, tryptamine, 5-hydroxytryptamine, N-acetylserotonin and MT in *Xoo* cells.

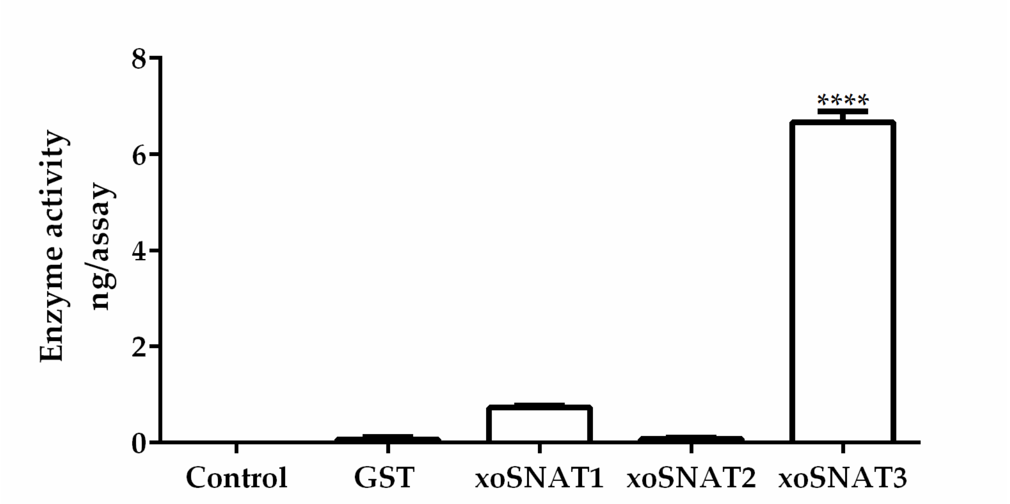


**Figure S2.** Enzymatic activity of GST, xoSNAT1, xoSNAT2 and xoSNAT3. The expression of GST, xoSNAT1, xoSNAT2 and xoSNAT3 in E. coli was induced by the addition of IPTG (0.4 mM) at 16 ºC for 16 h, respectively. These proteins were purified by using affinity chromatography column. Next, 1 μg of purified protein was incubated with serotonin (0.5 mM) at 35 ºC, the serotonin and product were both determined by LC-MS/MS. Control means enzymatic activity of 100 μL reaction buffer (0.5 mM serotonin, 0.5 mM acetyl-CoA and 100 mM potassium phosphate, pH = 8.8) in the absent of GST-

xoSNAT3. Asterisks indicate statistically significant differences determined using Student's t-test (**** $p < 0.0001$).
